# Endoscopic transorbital approach with electrocorticography for resection of anterior temporal lobe mass

**DOI:** 10.3171/2025.1.FOCVID24196

**Published:** 2025-04-01

**Authors:** Ruju Talati, Michael T. Walsh, Alex Schneider, Stacy Scofield-Kaplan, Osaama Hassan Khan

**Affiliations:** Department of Neurosurgery, Northwestern Medicine, Central DuPage Hospital and Cancer Center, Winfield, Illinois

**Keywords:** pilocytic astrocytoma, seizures, minimally invasive, lateral orbitotomy, orbital osteotomy, middle fossa

## Abstract

The authors present the case illustration of a 32-year-old female with worsening seizures with slight right orbital proptosis of unclear etiology. Imaging revealed a growing right temporal pole mass concerning for a glial tumor. The patient was an ideal candidate for transorbital endoscopic resection given its location and her epilepsy history. The mass was completely removed with the aid of electrocorticography (ECOG) to ensure no additional perilesional epileptic focus. The patient has had a 6-month follow-up with no worsening seizures and favorable cosmetic outcome.

The video can be found here: https://stream.cadmore.media/r10.3171/2025.1.FOCVID24196

## Transcript

### 0:26 Case Presentation.

This is a 32-year-old female with a past medical history of partial complex seizures since age of 18 who was referred to neurosurgery clinic for a change in semiology with increase in frequency and duration of partial complex seizures. Her exam was remarkable for slight right proptosis. Prior imaging shows a 2004 CT scan of a right middle cranial fossa hypodensity that on repeat imaging in 2011 and 2014 was stable. An MRI from that time was not available.

### 0:56 Magnetic Resonance Imaging.

New MRI was obtained which shows an enhancing right temporal pole with no surrounding vasogenic edema.

### 1:07 Clinical Decision.

Patient’s case was discussed at multidisciplinary rounds. Patient was offered medical management versus a surgical intervention. The transorbital would allow for a smaller scar, direct approach to the pathology, and less brain manipulation^1^ while still obtaining ECOG intraoperative testing.

### 1:23 OR Setup.

Our endoscopic transorbital approach involves two IVs, arterial line, Foley, neuromonitoring for ECOG, antiepileptic as per neurology, neuronavigation, and Mayfield pins in neutral position. We routinely do not use lumbar drain or fat graft.^2–4^

### 1:38 Skin Marking and Local Anesthetic.

Lateral upper eyelid crease was marked. Silastic corneal shield with lubricant placed. Skin and periosteum was infiltrated with 1% lidocaine with 1:100,000 epinephrine.

### 1:52 Skin Incision.

Fifteen blade was used to cut through skin and orbicularis muscle. Preseptal dissection was used followed by monopolar to then get to the subperiosteal dissection. After the lateral orbital rim has been isolated, fishhooks are applied. We like to instrument the bone prior to the orbitotomy.

### 2:10 Orbitotomy and Sphenoid Drilling.

A sagittal or oscillating saw is used with the protection of the globe and temporalis muscle.

An osteotome can be used to facilitate a cosmetic fracture, with a rongeur ultimately used to outfracture the bone. Further drilling is performed with a high-speed drill with copious irrigation until the dura is appreciated. We like to use dynamic retraction at times when we can avoid retraction of the globe. Kerrison punches can be used to help complete the exposure, and the dura can be appreciated in the depth. Hemostasis is achieved prior to dural opening. Neuronavigation is used to confirm localization of the dural opening.

### 3:11 Opening of Dura.

A C-shaped incision was marked out with the bipolar, and an 11 blade was used to perform the dural opening with microscissors.

As the dura is opened, you can appreciate the normal brain and the vessel in the sulcus that separates the tumor from the surrounding brain.

### 3:39 Electrocorticography.

A four-contact platinum strip is then placed in different directions for ECOG testing prior to tumor removal. These results were unremarkable. For the surgical approach, resection and closure and endoscope is usually used; however, for illustrative purposes the microscope was used for publication purposes. Using standard microsurgical techniques, the tumor is coagulated and we define the plane between the tumor and the sulcus. Microscissors used for sharp dissection. Frozen was sent off, confirming a glial tumor. The goal of the surgical approach was to isolate the tumor, to circumscribe it, and to define the anatomical landmarks. The medial superior plane was identified, and the arachnoid was coagulated and cut, and a patty was placed. Further debulking of the tumor to aid in manipulation and to define anatomical landmarks. Oculoplastics routinely removed any malleable retractors and visualized the pupil to ensure no prolonged retraction. The tumor was soft and had a purplish appearance. It was not vascular. It was easy to define the plane between the brain and the tumor. Here we can see us manipulating it away and further debulking.

The tumor was then manipulated and lateralized, and you can see the medial white matter that is showing that we are around the tumor. White matter can be appreciated now in the depth as the tumor is manipulated with a ring curette.

Once the tumor is completely free, the last amount of tumor is removed. Further inspection is performed to ensure no residual. Copious irrigation is used and hemostasis achieved.

### 6:50 Repeat ECOG.

Postoperative ECOG is then performed in various directions and in the cavity to ensure no epileptic activity. In the future, we could consider placing the strip far around the anterior temporal tip and also consider attempting to reach the hippocampus.

### 7:18 Reconstruction and Closure.

Reconstruction and closure is performed. 4-0 Nurolon is used to reapproximate the dura. Gelfoam and Surgicel is placed over the dural opening, followed by fibrin glue and a collagen matrix dural substitute. We only use fat as an overlay when there is a large dural defect like in meningioma resections, but it is not unreasonable to place for a multilayer closure. Further fibrin glue is placed. The lateral orbital rim is placed back with 4-mm screws. The periosteum and temporalis muscle was reapproximated with interrupted 3-0 Vicryl. 6-0 Prolene was then used in a running fashion to close the skin. The Prolene sutures are removed in oculoplastics clinic on postoperative day 5.

### 8:42 Postoperative Imaging and Pathology.

CT scan 3D reconstruction shows lateral orbital plating and greater sphenoid wing drilling. Top three panels show preoperative CT, and the bottom three, postoperative. Postoperative MRI on the bottom three panels shows gross-total resection. Final pathology shows a pilocytic astrocytoma WHO grade 1.^5^

### 9:06 Postoperative Course.

Postoperative day 10 clinic visit shows normal extraocular movements. Six-month follow-up visit shows no seizures, no change in antiepileptics, no recurrence on MRI, and improvement in proptosis.

## Disclosures

The authors report no conflict of interest concerning the materials or methods used in this study or the findings specified in this publication.

## Author Contributions

Primary surgeon: Khan. Assistant surgeon: Scofield-Kaplan. Editing and drafting the video and abstract: Khan, Talati, Walsh, Scofield-Kaplan. Critically revising the work: Khan, Walsh, Scofield-Kaplan. Reviewed submitted version of the work: Khan, Walsh, Schneider, Scofield-Kaplan. Approved the final version of the work on behalf of all authors: Khan.

## Supplemental Information

### Patient Informed Consent

The necessary patient informed consent was obtained in this study.
